# P-2119. Low Incidence of Invasive Fungal Infections in Pre-Transplant and Transplant-Ineligible Multiple Myeloma Patients: A U.S. Claims-Based Analysis (2017-2021)

**DOI:** 10.1093/ofid/ofaf695.2283

**Published:** 2026-01-11

**Authors:** Daniel Rogers, Aubrey Baker, Jianing Xu, Xianyan Chen, Andrés F Henao Martínez, Daniel B Chastain

**Affiliations:** Emory Decatur Hospital, Lawrenceville, GA; Grady Health System, Atlanta, Georgia; University of Georgia, Athens, Georgia; UGA Franklin College of Arts and Sciences, Athens, Georgia; University of Colorado Anschutz Medical Campus, Aurora, Colorado; University of Georgia College of Pharmacy, Albany, GA

## Abstract

**Background:**

Invasive fungal infection (IFI) incidence and risk factors in multiple myeloma (MM) are poorly defined due to heterogeneous study populations. This study aimed to determine IFI incidence and risk factors for IFIs in pre-transplant or transplant-ineligible MM patients.

**Figure:**
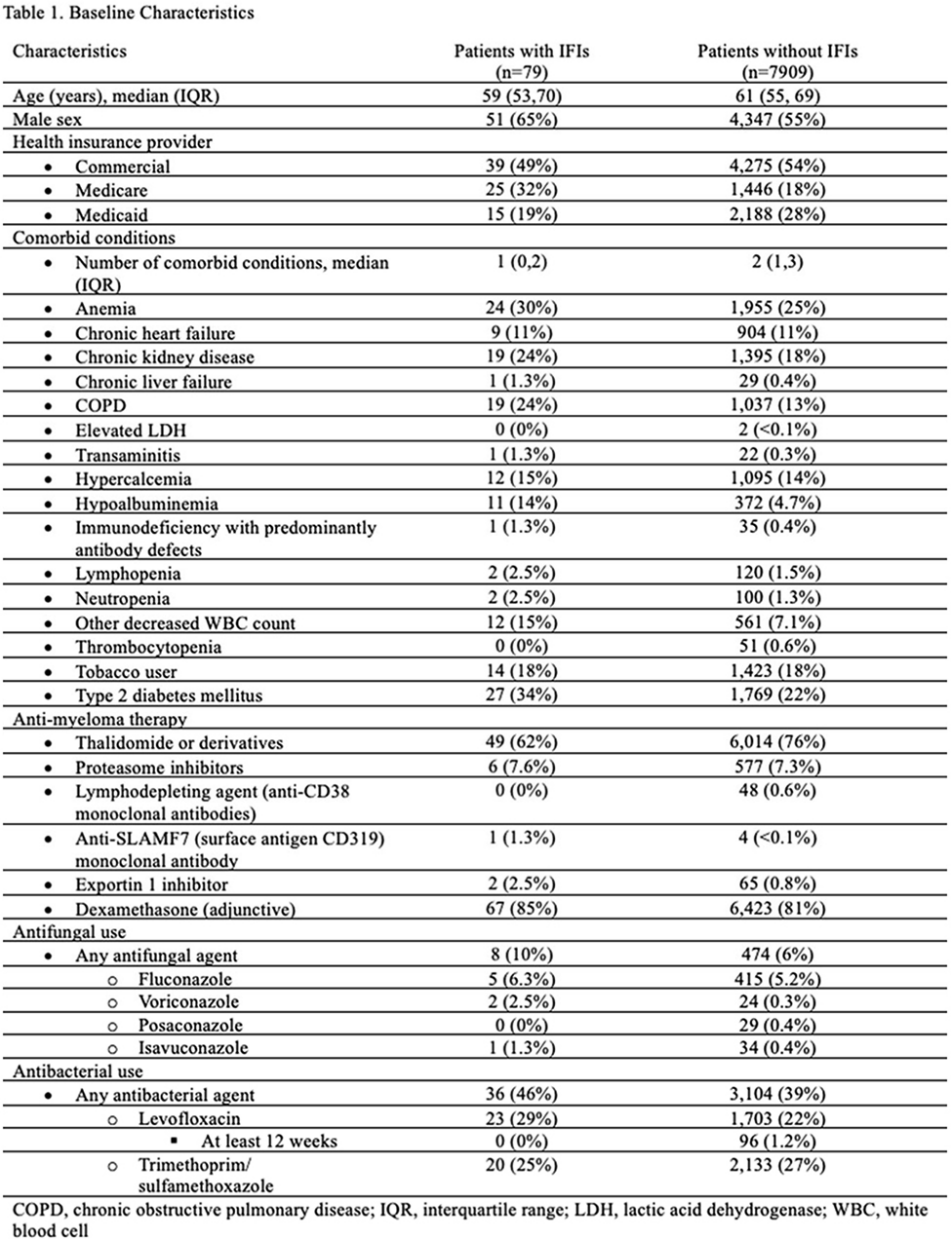

**Figure:**
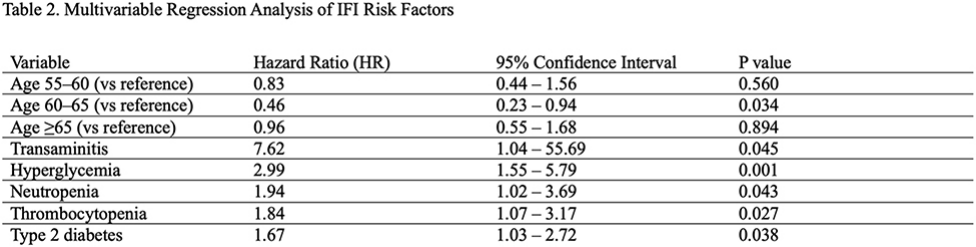

**Methods:**

We analyzed the MarketScan Database (2017-2021) to identify adults (≥18 years) diagnosed with and treated for MM, excluding those receiving prior anti-myeloma therapy within 5 months using a 6-month washout. Patients were followed from anti-myeloma therapy initiation until IFI diagnosis or censoring due to loss to follow-up or hematopoietic cell transplant. We assessed dexamethasone, antimicrobial, and anti-myeloma therapy use within 30 and 90 days before IFI diagnosis. IFI incidence and risk factors were determined, including clinical characteristics and treatment exposures. A case-control study (1:2 ratio) matched cases to controls by sex, age (±5 years), and follow-up period to assess IFI risk factors in the 1- and 3-month periods before IFI diagnosis or censoring.

**Figure:**
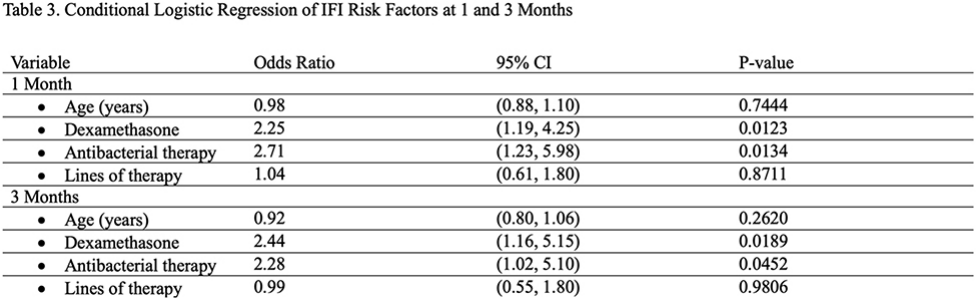

**Figure:**
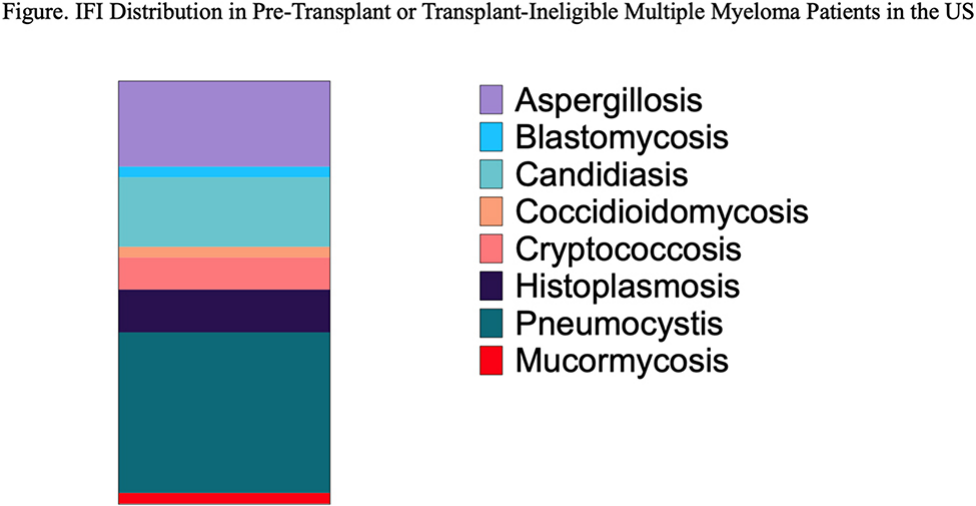

The stacked column graph illustrates the distribution of fungal infections among 79 pre-transplant or transplant-ineligible multiple myeloma patients in the United States. Pneumocystis was the most frequently reported infection (30 cases, 38%), followed by aspergillosis (16 cases, 20%), candidiasis (13 cases, 16%), and histoplasmosis (8 cases, 10%). Less common infections included cryptococcosis (6 cases, 7.6%), blastomycosis, coccidioidomycosis, and zygomycosis (each with 2 cases, 2.5%). No cases of paracoccidioidomycosis or sporotrichosis were identified.

**Results:**

Among 7988 patients with MM, 79 (< 1%) were diagnosed with IFIs, most commonly pneumocystis (38%) and aspergillosis (20%) (Fig). IFI patients were slightly younger and more often male (Table 1). COPD (24% vs 13%), chronic kidney disease (24% vs 18%), and type 2 diabetes (34% vs 22%) were more prevalent in the IFI group. Thalidomide derivatives were less frequent in IFI patients (62% vs 76%), while dexamethasone was slightly more common (85% vs 81%). Cox regression identified increased IFI risk with transaminitis (HR 7.62, p=.045), hyperglycemia (HR 2.99, p=.001), neutropenia (HR 1.94, p=.043), thrombocytopenia (HR 1.84, p=.027), and type 2 diabetes (HR 1.67, p=.038) (Table 2). Conditional logistic regression indicated increased IFI risk with glucocorticoid use within 1 and 3 months prior to IFI diagnosis (Table 3).

**Conclusion:**

The overall IFI incidence is low in pre-transplant and transplant-ineligible MM patients, but IFI occurrence is driven by both disease-related factors and treatment-related toxicities. Specifically, pre-existing type 2 diabetes, treatment complications (transaminitis, hyperglycemia, and hematologic cytopenias), and glucocorticoid use identify patients at increased risk.

**Disclosures:**

Andrés F. Henao Martínez, MD, MPH, F2: Grant/Research Support|Scynexis: Grant/Research Support

